# Dose response of umeclidinium administered once or twice daily in patients with COPD: a randomised cross-over study

**DOI:** 10.1186/1471-2466-14-2

**Published:** 2014-01-06

**Authors:** Alison Church, Misba Beerahee, Jean Brooks, Rashmi Mehta, Palvi Shah

**Affiliations:** 1 Respiratory Medicines Development Center, GlaxoSmithKline, Research Triangle Park, NC 27709, USA; 2 Clinical Pharmacology Modelling & Simulation, GlaxoSmithKline, Stevenage SG1 2NY, UK; 3 Respiratory Medicines Development Centre, GlaxoSmithKline, Stockley Park, Uxbridge UB11 1BT, UK; 4 Research & Development, GlaxoSmithKline, Stockley Park, Uxbridge UB11 1BT, UK

**Keywords:** Chronic obstructive pulmonary disease (COPD), Long-acting bronchodilators, Long-acting muscarinic antagonist (LAMA), Umeclidinium (UMEC), GSK573719

## Abstract

**Background:**

Umeclidinium bromide (UMEC) is an inhaled long-acting muscarinic antagonist in development for chronic obstructive pulmonary disease (COPD).

**Methods:**

This was a multicentre, randomised, double-blind, placebo-controlled, three-way cross-over, incomplete block study to evaluate UMEC 15.6, 31.25, 62.5, and 125 μg administered once daily (QD), and UMEC 15.6 μg and 31.25 μg administered twice daily (BID), over 7 days in patients with COPD. Tiotropium was included as an open-label treatment arm. The primary efficacy endpoint was trough forced expiratory volume in 1 second (FEV_1_) on Day 8. Secondary efficacy endpoints included weighted mean FEV_1_ over 0–24 hours after morning dosing on Day 7, and serial FEV_1_ at each time point over 24 hours after morning dosing on Day 7. Safety and pharmacokinetics were also examined.

**Results:**

One hundred and sixty-three patients (mean age 59.5 years, 52% female) were randomised. Based on the population dose–response model of trough FEV_1_ data, the geometric mean potency (ED_50_) of UMEC was 37 μg (95% confidence interval [CI]: 18, 57) with a predicted maximum intrinsic efficacy (E_max_) at trough of 0.185 L (95% CI: 0.153, 0.218) after QD dosing. UMEC 125 μg QD demonstrated the greatest improvements in measure of lung function compared with doses of 62.5 μg and below. UMEC 125 μg QD exhibited more consistent increases in FEV_1_ from baseline across serial time points over 24 hours compared with other UMEC doses and tiotropium. Increases in FEV_1_ over 0–12 hours were similar to those observed over 12–24 hours after the second dose of UMEC was administered. UMEC was rapidly absorbed following inhaled dosing and eliminated from plasma. Adverse events, generally mild, were highest with UMEC 125 μg QD (18%) compared with placebo (8%), tiotropium (4%) and other UMEC doses (5–12%).

**Conclusions:**

UMEC is a potent QD bronchodilator with geometric mean ED_50_ of 37 μg. A dose ordering over the range of UMEC 15.6–125 μg QD doses was observed, with UMEC 125 μg showing the greatest improvement in trough FEV_1_.

**Trial registration:**

GlaxoSmithKline funded (clinicaltrials.gov NCT01372410; GlaxoSmithKline study number AC4115321).

## Background

Cholinergic tone is considered the major reversible component of airflow obstruction in patients with chronic obstructive pulmonary disease (COPD) [1]. Receptor antagonism by antimuscarinic agents facilitates relaxation of airway smooth muscle. These agents bind to muscarinic receptor subtypes M_1_ and M_3_ localised in airway smooth muscle and block the bronchoconstrictor response to cholinergic nerve stimulation [2,3], thereby improving airflow obstruction. Long-acting muscarinic antagonists (LAMAs) have been shown to be a more effective and convenient treatment for COPD than short-acting bronchodilators [4].

Umeclidinium bromide (UMEC; GSK573719) is an inhaled LAMA in development for the treatment of COPD. Pharmacology studies have demonstrated that single- and repeat-dose UMEC administration is well tolerated in healthy volunteers [5] and in patients with COPD [6,7]. Statistically significant improvements in change from baseline in trough forced expiratory volume in 1 second (FEV_1_) were demonstrated compared with placebo [6,8]. Although the dose–response curve of UMEC from 62.5 μg to 1000 μg has been examined over 14 [6] and 28 days [8], the response obtained over this dosing range was relatively flat.

The current study examined the dose–response and safety of UMEC 15.6, 31.25, 62.5, and 125 μg administered once daily (QD), and UMEC 15.6 and 31.25 μg twice daily (BID). A model-based approach to the assessment of dose response has the advantage of utilising information within and across the range of doses studied for a more informed assessment of the drug’s dose–response relationship [9]. Preliminary results have been presented in abstract form [10].

## Methods

### Patients

Study investigators enrolled eligible patients who were 40–80 years of age, had a clinical history of COPD as defined by the American Thoracic Society (ATS)/European Respiratory society [11], were current or former cigarette smokers with a history of cigarette smoking of ≥10 pack-years, and had a post-salbutamol FEV_1_/forced vital capacity (FVC) ratio of <0.70 and a post-salbutamol FEV_1_ of ≥35% and ≤70% of predicted normal values [12,13]. Patients were excluded if they had a current diagnosis of asthma, known α1-antitrypsin deficiency, active lung infections, lung cancer, any clinically significant uncontrolled disease, or an abnormal and significant electrocardiogram (ECG) or significantly abnormal clinical laboratory finding. Concomitant use of inhaled salbutamol as a rescue medication was allowed. Inhaled corticosteroids (ICS) at a dose up to 1000 μg/day of fluticasone propionate or equivalent were permitted, provided the dose remained stable throughout the treatment period. Initiation or discontinuation of ICS or long-acting β_2_-agonist/ICS combinations within 30 days prior to screening was prohibited; however, patients were allowed to discontinue long-acting β_2_-agonist/ICS up to 2 days prior to screening if ICS alone was continued.

Written consent was obtained prior to the start of study-specific procedures. The study (clinicaltrials.gov NCT01372410; GSK study number AC4115321) was approved by the local ethics review committee (Chesapeake IRB, Columbia, MD) and was conducted in accordance with the Declaration of Helsinki and Good Clinical Practice guidelines [14].

### Study design and treatment

This randomised, incomplete block, three-period cross-over, placebo-controlled study was conducted in 15 centres in the United States from 25 July 2011 to 27 October 2011. In accordance with the randomisation schedule, generated using SAS and RandAll version 2.5, patients were assigned to receive a sequence of three of eight potential treatments for a total of three treatment periods per patient. UMEC 15.6 μg QD, 31.25 μg QD, 62.5 μg QD, 125 μg QD, 15.6 μg BID, 31.25 μg BID, open-label tiotropium 18 μg QD, and placebo were evaluated. UMEC and matching placebo study medication were administered via the ELLIPTA™ dry powder inhaler ^a^ in a double-blind fashion where neither patients nor the study investigators knew which study medication was administered. Tiotropium was an open-label comparator administered via the Handihaler® ^b^. To maintain blinding, patients taking QD treatments also took placebo in the evening. Treatment consisted of three 7-day treatment periods, with two intervening 10–14 day washout periods. A 7–9 day washout period followed the third treatment period, before a follow-up phone call.

Study withdrawal criteria included COPD exacerbation (defined as acute worsening of COPD symptoms requiring treatment beyond study medication or rescue salbutamol, including antibiotics or systemic corticosteroids, and/or hospitalisation or emergency treatment), clinically significant change in laboratory parameters, or elevated liver chemistry.

Treatment adherence was assessed on Day 7 of each treatment period using dose counters on the inhaler or by counting blister doses remaining (tiotropium).

### Outcomes and assessments

The primary efficacy endpoint was trough FEV_1_ on Day 8, defined as the mean of the FEV_1_ values obtained 23 and 24 hours after morning dosing on Day 7 of each treatment period.

The secondary efficacy endpoints were weighted mean FEV_1_ over 0–24 hours after morning dosing on Day 7, and serial FEV_1_ over 24 hours after morning dosing on Day 7. Serial spirometry was measured 30 and 5 minutes pre-morning dose, 1, 3, 6, 9, 12 (pre-evening dose), 13, 15, 23 and 24 hours after morning dosing.

Additional efficacy endpoints included trough FEV_1_ at other time points, weighted mean FEV_1_ over other time periods, serial FEV_1_, trough FVC, weighted mean FVC, and rescue salbutamol use (mean number of puffs per day and percentage of rescue-free days). Safety assessments included incidence of adverse events (AEs), haematology and clinical chemistry evaluations, incidence of COPD exacerbations, and vital signs.

Plasma and urine samples were collected for pharmacokinetic (PK) analysis. Assessments included area under concentration-time curve from time 0 to time t (AUC_(0-t)_), maximum observed plasma concentration (C_max_), time of maximum observed plasma concentration (t_max_), amount of drug excreted unchanged in urine, and fraction of dose excreted unchanged in urine. Accumulation was calculated using plasma C_max_, plasma AUC using a common sampling time and amount excreted in urine over the same time interval on Day 7 versus Day 1.

### Spirometry measurements

Measurements for FEV_1_ and FVC were obtained using standard spirometry equipment that met or exceeded the minimal ATS performance recommendations [15]. Spirometry was performed at screening, during a 6-hour interval on the first day of each treatment period, and during a 24-hour interval on the last day of each treatment period. A minimum of three acceptable spirometry efforts were obtained for FEV_1_ and FVC, and the highest measurement was recorded. Pre- and post-salbutamol spirometry measures at screening determined patient eligibility.

### Sample size determination

The sample size for the population model-based dose response analysis was determined using the Monte-Carlo Mapped Power approach for mixed effects [16]. The dose response from a UMEC dose-ranging study was used as reference [6]. Using a model-based approach for a cross-over design study, 16 patients would provide at least 90% power to show a significant dose response with 25 patients showing ~95% power. The sample size was increased to reduce the risk of a false positive result for the lower UMEC doses – a sample size of 40 patients per arm would provide <10% chance that lower doses of UMEC would falsely show a trough FEV_1_ response of >100 mL. This number of patients would also provide approximately 85% power for the comparison of active treatments with placebo for the primary efficacy endpoint on Day 8 (ANCOVA analysis). This calculation assumes a two-sided 5% significance level, a within-patient standard deviation of 0.170 L (based on Donohue et al. [6]) and a treatment difference from placebo of 0.130 L. Therefore, approximately 160 patients were recruited to compensate for a possible 30% dropout rate.

### Study population

The primary population for all efficacy and safety analyses was the modified intent-to-treat (mITT) population, comprising all patients who were randomised and received at least one dose of study medication. The population was modified in that outcomes were analysed based on the actual treatment received rather than the randomised treatment. The PK population comprised all patients in the mITT population for whom a PK sample was obtained and analysed.

### Model-based and statistical analysis

Two approaches were used to characterise the relationship between dose and trough FEV_1_. The first approach (i.e., the primary analysis) was a model-based analysis whereby an E_max_ model was selected from a suite of dose response shape models to describe the observed trough FEV_1_ data as a function of dose. Two key parameters were estimated from this model - E_max_ which is an estimate of the maximum response predicted by the model given the observed trough FEV_1_ and ED_50_ which represents the dose that achieves 50% of E_max_. The modelling approach investigated the impact of the inter-patient variability in trough FEV_1_ by examining the influence of patient demographic and physiologic factors (Additional file 1), and the effect of QD and BID regimen on the model parameters. Established model diagnostics were derived [17,18] to demonstrate the suitability of the chosen dose–response model. Using the population E_max_ model, the predictive distribution of trough FEV_1_ across treatments was derived by simulating 1000 sets of individual model parameters using the covariance matrix (model uncertainty and random effects) of parameter estimates from the model. Key outputs included median trough FEV_1_ (95th percentiles) for QD and BID regimens, the probability of achieving a certain target trough FEV_1_ with each dose (adjusted for baseline and placebo), and median estimates of trough FEV_1_ (adjusted for baseline and placebo) across the dose range and by dose regimen. Both mean baseline FEV_1_ and period were included as covariates.

A Day 8 dataset and a pooled dataset for Days 7 and 8 (*post-hoc* analysis) were analysed and reported for the primary efficacy analysis. The rationale for pooling Day 7 and Day 8 was to ensure informative interpretation of FEV_1_ response as function of dose, given the repeated measures for trough FEV_1_ response within each patient on different days.

The second approach (also referred to as the secondary analysis) involved comparison of trough FEV_1_ at each dose versus placebo using Analysis of Covariance (ANCOVA). The change from baseline FEV_1_ (defined as the mean of the two pre-morning dose assessments at Day 1) to trough FEV_1_ at Day 8 was analysed using a mixed model which included period baseline FEV_1_, mean baseline FEV_1_, treatment and period as fixed effects, and patient as a random effect. A similar methodology was used to analyse weighted mean FEV_1_ and trough and weighted mean FVC endpoints. Serial FEV_1_ was analysed using a similar mixed model. Sensitivity analyses were conducted to assess the effect of any interaction of treatment with mean baseline, period baseline or period by repeating the analysis of trough FEV_1_ on Day 8 and adding a variable to indicate the previous treatment received, removing baselines from the model or both.

Due to issues with Good Clinical Practice at one investigator site, a decision was made after unblinding to re-evaluate the dose–response model and ANCOVA analysis of trough FEV_1_ on Day 8, excluding all patients enrolled at that site.

A Bayesian analysis of the primary endpoint (using the same covariates as the original mixed- model analysis) provided the posterior probability distribution of the treatment difference of each treatment against placebo, i.e. the distribution of the true treatment difference given the data observed in the study.

## Results

### Study population

**Figure 1:**
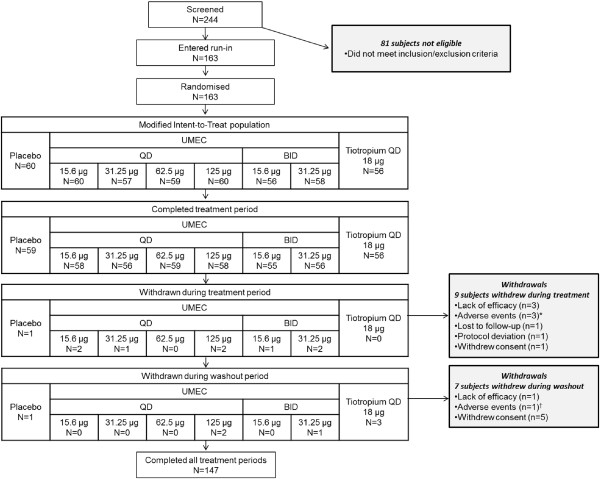
**Patient disposition and CONSORT flow chart.** *1 patient each in UMEC 15.6 μg QD (acute respiratory failure), UMEC 31.25 μg QD (myocardial infarction), and UMEC 31.25 μg BID (dizziness) during active treatment. ^†^1patient in UMEC 125 μg QD (nasopharyngitis) during washout. Number of patients (*N*) for completed treatment period, withdrawn during treatment period, and withdrawn during washout period reflects the total number of patients in each treatment group across all three treatment periods who completed the treatment period, withdrew during the treatment period, or withdrew during the washout period; two patients included in the counts for ‘withdrawn during a treatment period’ and not included in the counts and percentages for ‘withdrawn during a washout period’ actually withdrew during a washout period. BID, twice daily; QD, once daily; UMEC, umeclidinium bromide.

Two hundred and forty-four patients were screened, 163 were randomised (mITT population; Figure 1), and 90% completed the study. Mean treatment compliance was ≥99.3% across all treatment periods.

Patient demographics and baseline clinical characteristics are summarised in Table 1. Overall, the study population had moderate to severely impaired airflow obstruction at screening, as evident by values for mean post-bronchodilator FEV_1_ and FEV_1_/FVC ratio, and mean post-salbutamol FEV_1_ and FVC. Across treatment groups, 20–32% of patients took concomitant COPD medication, most often ICSs (18–30%) followed by oxygen (4–8%).

**Table 1:** Summary of patient population (mITT population), (a) demographic characteristics (b) screening parameters

**a.**		
**Characteristic**	**Total**	
	***N*** **= 163**	
Age (yr)		
Mean (SD)	59.5 (9.21)	
Sex, *n* (%)		
Male	78 (48)	
Ethnicity, *n* (%)		
Hispanic/Latino	1 (<1)	
Non-Hispanic/Latino	162 (>99)	
Race, *n* (%)		
White	145 (89)	
African American/African heritage	16 (10)	
African American/African heritage and White	1 (<1)	
American Indian or Alaskan native	1 (<1)	
Height (cm)		
Mean (SD)	170.2 (9.20)	
Weight (kg)		
Mean (SD)	79.55 (17.539)	
Body mass index (kg/m^2^)		
Mean (SD)	27.36 (5.115)	
**b.**		
**Parameter**	**Total *N*** **= 163**	
	**Pre-salbutamol**	**Post-salbutamol**
% predicted FEV_1_ (%)		
*n*	162	163
Mean (SD)	47.0 (12.84)	51.1 (10.16)
FEV_1_/FVC (%)		
*n*	162	163
Mean (SD)	51.1 (11.65)	52.3 (10.62)
FEV_1_ (L)		
*n*	162	163
Mean (SD)	1.429 (0.5179)	1.554 (0.4727)
FVC (L)		
*n*	162	163
Mean (SD)	2.803 (0.7948)	3.001 (0.8073)
Reversibility to salbutamol (%)		
*n*	162	
Mean (SD)	11.8 (15.31)	
Reversibility to salbutamol (mL)		
*n*	162	
Mean (SD)	124.2 (212.56)	
Mean baseline FEV_1_ (L)		
*n*	163	
Mean (SD)	1.408 (0.5282)	
Mean baseline FVC (L)		
*n*	163	
Mean (SD)	2.763 (0.7920)	

Note: mean baseline was defined as the mean of the baseline values from each treatment period. If one or more values were missing, the mean baseline was the mean of the non-missing values.

FEV_1_, forced expiratory volume in 1 second; FVC, forced vital capacity; mITT, modified intent-to-treat; SD, standard deviation; UMEC, umeclidinium bromide.

### Efficacy

#### Final dose–response model

The physiological E_max_ model (see Additional file 2) was optimal in defining the relationship between UMEC doses and the primary endpoint of trough FEV_1_ on Day 8; the parameters of the model were estimated with high precision (coefficient of variation <30%). The ED_50_ for UMEC QD dosing was 37 μg (95% CI: 18–57). The predicted E_max_ was 0.185 L (95% CI: 0.153, 0.218). Model diagnostics show suitability of the final model in describing the dose–response relationship (see Additional file 3). Pooled data from Day 7 and Day 8 were similar: ED_50_ of 38 μg and predicted E_max_ of 0.156 L.

**Figure 2:**
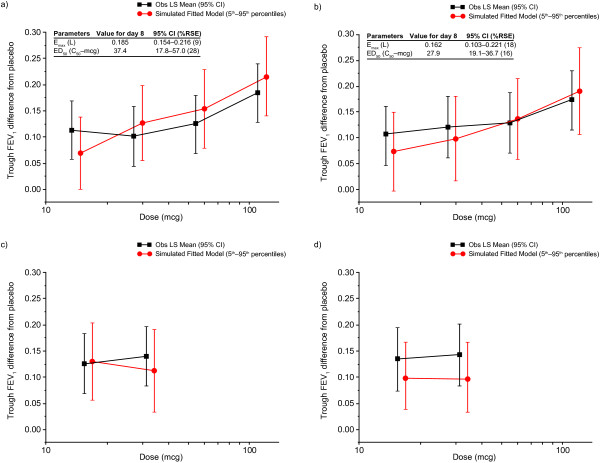
**Observed LS mean trough FEV**_**1 **_**and simulated fitted model for UMEC QD and BID regimens.** Data are for **(a)** UMEC QD in the mITT population, **(b)** UMEC QD in the mITT population excluding patients from one investigator site, **(c)** UMEC BID in the mITT population, and **(d)** UMEC BID in the mITT population excluding patients from one investigator site. CI, confidence interval; ED_50_ (C_50_), dose that yields 50% of E_max_; E_max_, maximum predicted FEV_1_ response; FEV_1_, forced expiratory volume in 1 second; Obs LS, observed least square; RSE, relative standard error.

The model-based simulated median estimates of FEV_1_ for the QD regimen were plotted together with the observed least square (LS) mean FEV_1_ treatment differences (ANCOVA analysis) (Figure 2a). Both methods showed reasonable agreement in the FEV_1_ response with dose with the model-based analysis showing a clear monotonic dose response. The dose–response curve suggests that trough FEV_1_ response did not plateau at the highest dose. Results of the *post-hoc* model analysis excluding one investigator site (due to issues of Good Clinical Practice) were generally consistent with those for the mITT population (Figure 2b). Similar trends were also observed for the BID regimen in the mITT (Figure 2c) and the *post-hoc* model analysis excluding one investigator site (Figure 2d).

Simulation based on the final dose–response model was used to estimate the probability of achieving a particular response at a given dose (Table 2); this application of the model utilised the integrated variability between- and within-patients across doses to provide a better insight into the dose–response relationship. Thus, UMEC 62.5 μg and 125 μg QD had a ≥87% probability of exceeding the minimum clinically relevant target of 0.100 L trough FEV_1_[6] and a ≥77% probability of exceeding a 0.120 L trough FEV_1_ (Table 2). Based on the total daily UMEC dose, the model-based analysis suggests that although there were small numerical differences between the 31.25 μg QD and 15.6 μg BID dosing regimens, the probability of exceeding the target response with the 31.25 μg BID dose is markedly lower than that for the 62.5 μg QD dose (Table 2).

**Table 2:** Probability of a dose exceeding a target response (adjusted for placebo) (mITT population)

**UMEC dose**	**Probability (%) of exceeding a specified change from baseline FEV**_**1 **_**at trough (Day 8 dataset)**			
	**0.075 L**	**0.100 L**	**0.120 L**	**0.150 L**
15.6 μg QD	43	21	11	3
31.25 μg QD	88	74	56	30
62.5 μg QD	95	87	77	52
125 μg QD	99	100	99	92
15.6 μg BID	89	75	59	33
31.25 μg BID	78	60	44	21

BID, twice daily; FEV_1_, forced expiratory volume in 1 second; QD, once daily; UMEC, umeclidinium bromide.

#### Trough FEV_1_ on Day 8 (ANCOVA analysis)

**Figure 3:**
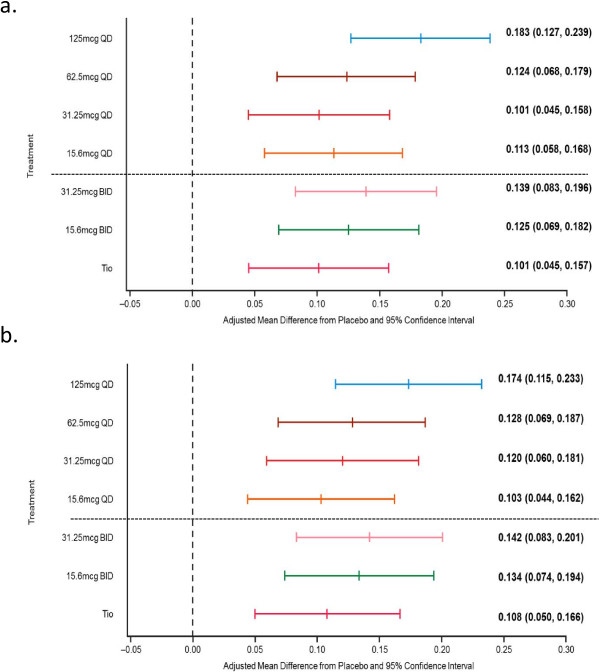
**Trough FEV**_**1 **_**(L) on Day 8 (mITT population) for UMEC QD and BID regimens.** Data are for **(a)** mITT population **(b)** mITT population excluding patients from one investigator site. BID, twice daily; QD, once daily.

Statistically significant increases in change from baseline in trough FEV_1_ over placebo on Day 8 were demonstrated for all UMEC QD and BID doses (range: 0.101–0.183 L; *p* < 0.001 for each comparison); tiotropium (0.101 L; *p* < 0.001) was within the range observed for UMEC doses (Figure 3). Dose ordering was observed across UMEC QD doses with UMEC 125 μg QD demonstrating the greatest improvements in trough FEV_1_. There was some evidence of an interaction of treatment with period baseline (*p* = 0.0014) and period (*p* = 0.0193). The conclusions from each sensitivity analysis performed to assess the impact of these interactions were similar to those from the pre-defined analysis and it was therefore concluded that any interaction with baseline was not significantly impacting study conclusions.

Bayesian analysis demonstrated that UMEC 62.5 μg and 125 μg QD showed high probabilities (80.1% and 99.8%, respectively) that the true treatment difference compared with placebo was greater than 0.100 L for trough FEV_1_ on Day 8. The probabilities for 15.6 μg and 31.25 μg QD were 68.2% and 52.0%, respectively. Additionally, UMEC 125 μg QD showed a 96.8% probability of having a true treatment difference from placebo of greater than 0.130 L as compared to tiotropium which showed a 15.9% probability.

#### 0–24-hour weighted mean FEV_1_ on Day 7

Statistically significant improvements in the adjusted mean changes from baseline in 0–24-hour weighted mean FEV_1_ over placebo on Day 7 were observed for all UMEC QD and BID doses and for tiotropium (Table 3). Dose ordering was observed across QD dose regimens with UMEC 125 μg QD showing the greatest improvements in 0–24-hour weighted mean FEV_1_.

**Table 3:** **0–24-hour weighted mean FEV**_**1 **_**(L) on Day 7 (mITT population)**

**0–24-hour weighted mean FEV**_**1 **_**(L)**	**Placebo *N*** **= 60**	**UMEC QD**			
		**15.6 μg**	**31.25 μg**	**62.5 μg**	**125 μg**
		***N*** **= 60**	***N*** **= 57**	***N*** **= 59**	***N*** **= 60**
*N*	54	56	51	54	56
LS mean (SE)	1.327 (0.018)	1.443 (0.018)	1.445 (0.019)	1.459 (0.018)	1.500 (0.018)
LS mean change (SE)	−0.074 (0.018)	0.043 (0.018)	0.045 (0.019)	0.059 (0.018)	0.100 (0.018)
Difference from placebo	NA	0.116	0.118	0.132	0.173
95% CI	NA	(0.072, 0.160)	(0.073, 0.163)	(0.087, 0.178)	(0.129, 0.217)
*p*-value	NA	<0.001	<0.001	<0.001	<0.001
**0–24-hour**	**UMEC BID**				**Tiotropium**
**weighted mean FEV**_**1 **_**(L)**	**15.6 μg**		**31.25 μg**		**QD 18 μg**
	***N*** **= 56**		***N*** **= 58**		***N*** **= 56**
*N*	52		55		53
LS mean (SE)	1.462 (0.018)		1.469 (0.018)		1.484 (0.018)
LS mean change (SE)	0.062 (0.018)		0.068 (0.018)		0.084 (0.018)
Difference from placebo	0.136		0.142		0.157
95% CI	(0.091, 0.181)		(0.098, 0.186)		(0.113, 0.202)
*p*-value	<0.001		<0.001		<0.001

Note: Analysis performed using a mixed model with covariates of trough mean baseline, trough period baseline, treatment and period as fixed effects and subject as a random effect.

For each treatment period, baseline was defined as the mean of the values obtained 5 and 30 minutes predose on Day 1.

BID, twice daily; CI, confidence interval; FEV_1_, forced expiratory volume in 1 second; LS, least square; mITT, modified intent-to-treat; NA, not applicable; QD, once daily; SE, standard error; UMEC, umeclidinium bromide.

#### Serial FEV_1_ over 24 hours on Day 7

**Figure 4:**
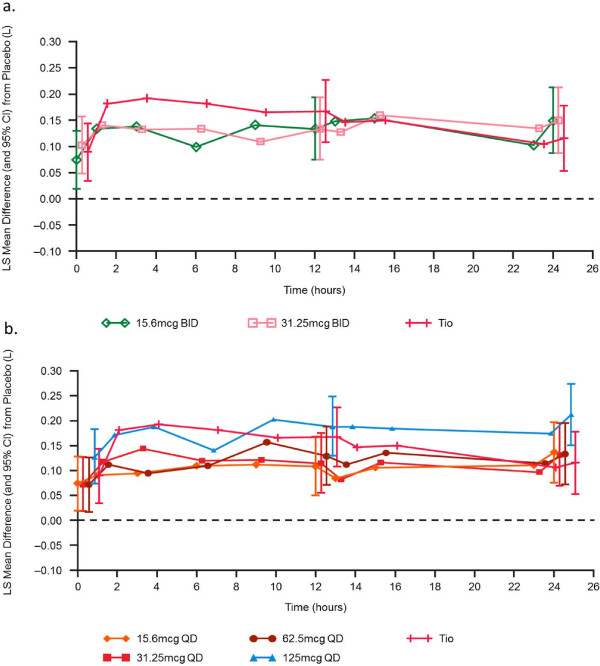
**Serial FEV**_**1 **_**on Day 7 adjusted treatment differences from baseline.** Data are for **(a)** UMEC QD and tiotropium, and **(b)** UMEC BID and tiotropium (mITT population). BID, twice daily; CI, confidence interval; LS, least square; QD once daily; Tio, tiotropium.

Serial FEV_1_ over time on Day 7 demonstrated statistically significant improvements in the adjusted mean changes from baseline over placebo for all UMEC doses and tiotropium at all time points (Figure 4). Increases were broadly consistent across all time points over 24 hours, with no marked improvement in FEV_1_ for either BID dose following a second UMEC dose at 12 hours. Improvements in FEV_1_ over placebo observed at time points over the first 12 hours were maintained over the second 12 hours for all UMEC QD doses, which is in contrast to an observed decline in FEV_1_ improvement at time points over the second 12 hours for tiotropium.

#### Trough FEV_1_ on Day 7

Statistically significant improvements in the adjusted mean changes from baseline in trough FEV_1_ over placebo on Day 7 were demonstrated for all UMEC QD and BID doses and for tiotropium (Table 4). The greatest improvement over placebo was observed for UMEC 125 μg QD (0.120 L). Improvements in trough FEV_1_ with UMEC compared with placebo on Day 7 were lower than improvements in trough FEV_1_ observed on Day 8, although a dose ordering was maintained with UMEC 125 μg QD providing the greatest improvement over both days.

**Table 4:** **Trough FEV**_**1 **_**(L) on Day 7 (mITT population)**

**Trough FEV**_**1 **_**(L)**	**Placebo**	**UMEC QD**			
		**15.6 μg**	**31.25 μg**	**62.5 μg**	**125 μg**
	***N*** **= 60**	***N*** **= 60**	***N*** **= 57**	***N*** **= 59**	***N*** **= 60**
*N*	60	59	56	59	60
LS mean (SE)	1.401 (0.019)	1.469 (0.019)	1.482 (0.020)	1.475 (0.019)	1.521 (0.019)
LS mean change (SE)	−0.014 (0.019)	0.054 (0.019)	0.067 (0.020)	0.060 (0.019)	0.106 (0.019)
Difference from placebo	N/A	0.068	0.081	0.074	0.120
95% CI	N/A	(0.018, 0.118)	(0.030, 0.131)	(0.024, 0.124)	(0.070, 0.170)
*p*-value	N/A	0.007	0.002	0.004	<0.001
**Trough FEV**_**1 **_**(L)**	**UMEC BID**				**Tiotropium QD**
	**15.6 μg**		**31.25 μg**		**18 μg**
	***N*** **= 56**		***N*** **= 58**		***N*** **= 56**
*N*	55		57		56
LS mean (SE)	1.484 (0.020)		1.500 (0.020)		1.493 (0.020)
LS mean change (SE)	0.069 (0.020)		0.085 (0.020)		0.078 (0.020)
Difference from placebo	0.083		0.099		0.092
95% CI	(0.032, 0.134)		(0.048, 0.149)		(0.041, 0.142)
*p*-value	0.001		<0.001		<0.001

Analysis performed using a mixed model with covariates of period baseline, mean baseline, treatment and period as fixed effects and patient as a random effect; for each treatment period, baseline was defined as the mean of the values obtained 5 and 30 minutes pre-dose on Day 1 and trough FEV_1_ is calculated from the FEV_1_ values obtained 23 and 24 hours after the Day 6 morning dose.

BID, twice daily; CI, confidence interval; FEV_1_, forced expiratory volume in 1 second; LS, least square; mITT, modified intent-to-treat; N/A, not applicable; QD, once daily; SE, standard error; UMEC, umeclidinium bromide.

#### 0–12-hour and 12–24-hour weighted mean FEV_1_ on Day 7

Improvements in the adjusted mean changes from baseline for UMEC QD doses in 12–24-hour weighted mean FEV_1_ over placebo were generally similar or greater than those observed for 0–12-hour weighted mean FEV_1_, indicating sustained duration of action (Table 5). The ratios of evening (12–24 hours) to morning (0–12 hours) weighted mean FEV_1_ values were approximately 1.0 for all UMEC QD and BID doses; whereas the ratio for tiotropium was approximately 0.8 (Table 6). Dose ordering was observed across the QD regimens with UMEC 125 μg having the largest effect in both 0–12-hour and 12–24-hour weighted mean FEV_1_.

**Table 5:** **0–12-hour and 12–24-hour weighted mean FEV**_**1 **_**(L) on Day 7, (a) morning and (b) evening dose (mITT population)**

**a.**					
**Morning dose:**		**UMEC QD**			
**0–12-hour**	**Placebo**	**15.6 μg**	**31.25 μg**	**62.5 μg**	**125 μg**
**weighted mean FEV**_**1 **_**(L)**	***N*** **= 60**	***N*** **= 60**	***N*** **= 57**	***N*** **= 59**	***N*** **= 60**
*N*	54	58	52	56	56
LS mean (SE)	1.353 (0.019)	1.467 (0.019)	1.478 (0.020)	1.481 (0.019)	1.526 (0.019)
LS mean change (SE)	−0.046 (0.019)	0.068 (0.019)	0.078 (0.020)	0.082 (0.019)	0.126 (0.019)
Difference from placebo	N/A	0.114	0.124	0.128	0.172
95% CI	N/A	(0.066, 0.161)	(0.075, 0.173)	(0.079, 0.177)	(0.124, 0.220)
*p*-value	N/A	<0.001	<0.001	<0.001	<0.001
**Morning dose:**	**UMEC BID**				**Tiotropium QD**
**0–12-hour**	**15.6 μg**		**31.25 μg**		**18 μg**
**weighted mean FEV**_**1 **_**(L)**	***N*** **= 56**		***N*** **= 58**		***N*** **= 56**
*N*	52		55		53
LS mean (SE)	1.483 (0.020)		1.483 (0.019)		1.530 (0.020)
LS mean change (SE)	0.084 (0.020)		0.084 (0.019)		0.130 (0.020)
Difference from placebo	0.130		0.130		0.176
95% CI	(0.081, 0.178)		(0.081, 0.178)		(0.128, 0.224)
*p*-value	<0.001		<0.001		<0.001
**b.**					
**Evening dose:**	**Placebo**	**UMEC QD**			
**12–24-hour**		**15.6 μg**	**31.25 μg**	**62.5 μg**	**125 μg**
**weighted mean FEV**_**1 **_**(L)**	***N*** **= 60**	***N*** **= 60**	***N*** **= 57**	***N*** **= 59**	***N*** **= 60**
*n*	54	56	52	54	56
LS mean (SE)	1.298 (0.020)	1.410 (0.020)	1.410 (0.020)	1.433 (0.020)	1.470 (0.020)
LS mean change (SE)	−0.103 (0.020)	0.010 (0.020)	0.010 (0.020)	0.032 (0.020)	0.069 (0.020)
Difference from placebo	N/A	0.112	0.112	0.135	0.172
95% CI	N/A	(0.064, 0.160)	(0.063, 0.162)	(0.085, 0.184)	(0.123, 0.221)
*p*-value	N/A	<0.001	<0.001	<0.001	<0.001
**Evening dose:**	**UMEC BID**				**Tiotropium QD**
**12–24-hour**	**15.6 μg**		**31.25 μg**		**18 μg**
**weighted mean FEV**_**1 **_**(L)**	***N*** **= 56**		***N*** **= 58**		***N*** **= 56**
*n*	52		55		53
LS mean (SE)	1.440 (0.020)		1.450 (0.020)		1.437 (0.020)
LS mean change (SE)	0.039 (0.020)		0.050 (0.020)		0.036 (0.020)
Difference from placebo	0.141		0.152		0.138
95% CI	(0.092, 0.191)		(0.103, 0.201)		(0.090, 0.187)
*p*-value	<0.001		<0.001		<0.001

**Table 6:** **Change from baseline in weighted mean FEV**_**1 **_**(L) difference in treatment effect compared between 12–24-hour and 0–12-hour at Day 7 (mITT population)**

	**Placebo**	**UMEC QD**			
		**15.6 μg**	**31.25 μg**	**62.5 μg**	**125 μg**
	***N*** **= 60**	***N*** **= 60**	***N*** **= 57**	***N*** **= 59**	***N*** **= 60**
*N*	54	58	53	56	56
Column vs. placebo	N/A	0.018	−0.007	0.021	0.010
Absolute difference					
95% CI	N/A	(−0.040, 0.076)	(−0.067, 0.053)	(−0.038, 0.080)	(−0.048, 0.069)
Ratio*	N/A	1.176	0.943	1.162	1.059
	**UMEC BID**				**Tiotropium QD**
	**15.6 μg**		**31.25 μg**		**18 μg**
	***N*** **= 56**		***N*** **= 58**		***N*** **= 56**
*N*	52		55		53
Column vs. placebo	0.019		0.028		−0.036
Absolute difference					
95% CI	(−0.041, 0.078)		(−0.030, 0.087)		(−0.095, 0.023)
Ratio*	1.147		1.215		0.793

*Relative to 0–12-hour weighted mean FEV_1_.

Absolute differences >0 indicate a larger treatment effect between 12–24 hours; analysis performed using a mixed model with covariates trough mean baseline, trough period baseline, treatment, period, time and time by treatment interaction as fixed effects and subject as a random effect.

Note: The column versus placebo difference was calculated as the difference in change from baseline in weighted mean FEV_1_ between active and placebo at 12–24 hours minus the difference in change from baseline in weighted mean FEV_1_ between active and placebo at 0–12 hours. The ratio is the difference in change from baseline in weighted mean FEV_1_ between active and placebo at 12–24 hours divided by the difference in change from baseline in weighted mean FEV_1_ between active and placebo at 0–12 hours.

BID, twice daily; CI, confidence interval; N/A, not applicable; QD, once daily; UMEC, umeclidinium bromide.

#### Trough and weighted mean on Day 7

Statistically significant increases in the adjusted mean changes from baseline in trough and 0–24-hour weighted mean FVC over placebo on Day 7 were demonstrated for all UMEC QD and BID doses. Improvements in trough FVC ranged from 0.128–0.181 L (*p* ≤ 0.006) for UMEC doses and 0.126 (*p* < 0.006) for tiotropium, and improvements in 0–24-hour weighted mean FVC ranged from 0.181–0.247 L (*p* < 0.001) for UMEC doses and 0.233 (*p* < 0.001) for tiotropium. Dose ordering was observed across QD doses for trough FVC and weighted mean FVC with UMEC 125 μg, showing the largest improvements compared with placebo. FVC values over 24 hours on Day 7 demonstrated statistically significant differences compared with placebo for all UMEC QD and BID doses and tiotropium.

#### Rescue salbutamol

Dose ordering was observed across UMEC QD doses for reductions in rescue salbutamol use compared with placebo which was consistent with the observed FEV_1_ response: UMEC 125 μg (0.804 puffs/day), 62.5 μg (0.464 puffs/day), 31.25 μg (0.283 puffs/day), and 15.6 μg (0.254 puffs/day). Tiotropium demonstrated a greater reduction in rescue salbutamol use compared with placebo (0.98 puffs/day) than those observed with UMEC. Increases from baseline in the percentage of rescue-free days demonstrated dose ordering: UMEC 125 μg (15.72%), 62.5 μg (12.50%), 31.25 μg (9.36%), and 15.6 μg (4.58%). Increases from baseline were also observed for tiotropium (11.32%).

### Pharmacokinetics

The plasma concentration profile for UMEC indicated that C_max_ occurred early (median t_max_ of 5–15 minutes) with doses above 31.25 μg QD showing a median t_last_ of 15 minutes, which was the last collected PK sample. The lower limit of UMEC quantification (10 pg/mL) and limited number of samples collected were not sufficient to fully characterise its PK profile; 46% of data were nonquantifiable. Comparison of UMEC QD and BID dosing regimens suggested that systemic exposure (i.e. AUC and C_max_) was higher following QD dosing than BID dosing for the same total daily dose. Following 7 days repeat dosing, the accumulation ratio ranged from 1.2 to 1.7 for UMEC systemic exposure. Approximately 2.2–2.5% of the total dose was excreted unchanged in urine in 24 hours after QD doses. Urine excretion of UMEC increased with dose, and the amount of UMEC excreted in the urine following Day 1 dose was lower than that following Day 7 dose for all dose levels.

### Safety

Overall incidence of AEs was low across treatments (4–18%). The most frequently reported AEs are shown in Table 7. Two serious AEs (acute respiratory failure, UMEC 15.6 μg QD and myocardial infarction UMEC 31.25 μg QD) were reported; neither was fatal or considered treatment-related by the investigator. Four COPD exacerbations were reported: one (placebo), two (UMEC 15.6 μg QD), and one (UMEC 125 μg QD).

**Table 7:** On-treatment adverse events reported by ≥3% of patients within any treatment group (mITT population)

	**Number (%) of patients**				
**Preferred term**		**UMEC QD**			
	**Placebo**	**15.6 μg**	**31.25 μg**	**62.5 μg**	**125 μg**
	***N*** **= 60**	***N*** **= 60**	***N*** **= 57**	***N*** **= 59**	***N*** **= 60**
Headache	2 (3)	1 (2)	0	0	3 (5)
Nasopharyngitis	0	1 (2)	0	0	1 (2)
Dysgeusia	0	1 (2)	0	0	2 (3)
Sinusitis	0	0	0	0	2 (3)
**Preferred term**	**UMEC BID**				**Tiotropium**
	**15.6 μg**		**31.25 μg**		**QD 18 μg**
	***N*** **= 56**		***N*** **= 58**		***N*** **= 56**
Headache	4 (7)		1 (2)		0
Nasopharyngitis	0		0		2 (4)
Dysgeusia	0		0		0
Sinusitis	0		0		0

Cut-off of ≥3% was based on percentage after rounding; on-treatment AEs were defined as AEs with onset within the period beginning with the first day of study drug administration through the day after the last day of study drug administration.

BID, twice daily; mITT, modified intent-to-treat; QD, once daily; UMEC, umeclidinium bromide.

UMEC was generally well tolerated at all doses, with no apparent dose-related changes in vital signs. Mean absolute values on Day 7 were similar across treatments for all clinical chemistry and haematology assessments except creatine kinase, which was elevated compared with placebo for UMEC 31.25 μg QD and BID and UMEC 62.5 μg QD. Most patients across all treatments had creatine kinase values within the normal range at baseline and any post-baseline assessment. The pattern of out of range values across UMEC QD treatments did not appear to be related to dose (8%, 13%, 9%, and 3% for the 15.6 μg, 31.25 μg, 62.5 μg, and 125 μg QD treatments, respectively, and 5% for placebo).

## Discussion

The current study demonstrated that QD dosing with UMEC provided statistically and clinically significant improvements in lung function over 24 hours. These results extend the findings from two previous dose-ranging studies [6,8] by demonstrating a dose ordering over a range of UMEC 15.6 to 125 μg QD and further substantiating that UMEC is efficacious when administered QD. In addition, trough FEV_1_ results for the overlapping doses of UMEC 125 μg QD and UMEC 62.5 μg QD from this study were similar to that observed in the previous dose-ranging studies (UMEC 125 μg QD: 0.147 L [95% CI: 0.077, 0.216] [6] and 0.159 L [95% CI: 0.088, 0.229] [8] and UMEC 62.5 μg QD: 0.128 L [95% CI: 0.060, 0.196] [6]).

Consistent with published dose–response models for bronchodilators [19,20], a population physiological E_max_ model characterised the relationship between UMEC dose and trough FEV_1_ on Day 8. A clear monotonic UMEC dose response was observed with dose ordering from the lowest (15.6 μg) to highest dose (125 μg). The maximum dose (125 μg UMEC QD) equated to approximately 3.4-fold ED_50_ of 37 μg estimated in this study. The trough FEV_1_ response for UMEC 125 μg QD (0.183 L) was near the maximum predicted response from the model (0.185 L) and was similar to the trough FEV_1_ response observed in the previous dose-ranging studies in patients with COPD, which ranged from 0.147 to 0.159 L [6,8].

A dose ordering was observed across UMEC QD dosing regimens with the UMEC 125 μg QD dose demonstrating the greatest improvement in lung function measurements compared with the lower QD doses. This pattern was further supported by Bayesian analysis of trough FEV_1_. Although all UMEC QD doses exhibited greater improvements in serial FEV_1_ measures compared with placebo at Day 7, UMEC 125 μg QD maintained greater increases from baseline in FEV_1_ across serial time points over 24 hours compared with the other QD doses.

Interestingly, greater improvements in trough FEV_1_ were observed on Day 8 compared to improvements observed on Day 7. This may be explained by the time of drug administration. Measurements on Days 7 and 8 were taken 23 and 24 hours after dosing; however, only dosing before the Day 8 measures was observed by study staff. Furthermore, patients were required to stay overnight between Days 7 and 8. Both of these factors may explain the differences observed in trough FEV_1_ across the two study days. Modelling of the pooled Day 7 and 8 data provided similar results to those on Day 8 only, and the ANCOVA results on Day 7 supported those on Day 8. Although patients with COPD are known to change their responsiveness to treatment over time, individual heterogeneity in response to treatment was not examined during this study, which may be viewed as a limitation. Individual response to treatment may have affected the interpretation of the study only because the study was small; however, the analysis was adjusted for the effect of patient, treatment and period.

The effects of UMEC QD versus BID dosing were also examined and BID regimens demonstrated small increases in the difference from placebo in some measures over QD regimens of the same total daily dose. Our study was not powered for formal comparisons between QD and BID doses. Given the small magnitude of improvement observed with BID dosing, it is considered unlikely to translate into a clinically significant benefit in efficacy. Improvements in serial FEV_1_ from placebo over the first 12 hours were maintained at time points over the second 12 hours for all UMEC QD doses, suggesting that QD dosing is supported for UMEC. Additionally, the ratios of evening (12–24-hour) to morning (0–12-hour) weighted mean FEV_1_ values were approximately 1.0 for all QD doses indicating that the 24-hour duration of effect is an intrinsic property of UMEC rather than being of a dose-related nature. The model-based analysis did not find dose regimen (QD versus BID) to be an important factor influencing dose response, which is also consistent with the previous study results [6]. This finding is not unexpected given that preclinical UMEC data demonstrated slow off-rate receptor kinetics in in-vitro human M3 receptor and bronchial tissue, and support a QD regimen [21]. It is noteworthy that the BID regimen showed small numerical increases in FEV_1_ values compared with those following QD and this finding is reflected in the probability values for 15.6 μg BID versus 31.25 μg OD. These numerical differences in FEV_1_ response between regimens are generally accounted for by the model-based analysis that integrated the variability estimates between- and within-patient across all doses [19]. Plausible reasons for the marked lower probability estimates at 31.25 μg BID compared with the 62.5 μg OD regimen may include study design factors, namely imbalance in patient dose allocation due to the incomplete block study design, or possible clinical under-performance of the 31.25 μg BID formulation.

The LAMA tiotropium was used as an active comparator in this study as it is a well-established medication for the treatment of COPD [22]. Although the observed improvement in trough FEV_1_ over placebo with tiotropium was within the range of those observed with UMEC doses, tiotropium was at the lower end of the range. Nearly all UMEC QD and BID doses demonstrated improvements over placebo in trough FEV_1_ on Day 8 (range: 0.101–0.183 L) that were numerically similar to or greater than tiotropium (0.101 L). However, trough FEV_1_ does not convey the full picture of bronchodilation offered to patients, and thus the 24-hour weighted mean and 24-hour serial FEV_1_ profiles were also evaluated. The 24-hour weighted mean and 24-hour serial FEV_1_ profiles showed UMEC doses of 62.5 and 125 μg were the most similar in efficacy to tiotropium, with the bronchodilator effect of the 62.5 μg dose generally less and the effect of the 125 μg dose generally greater than that of tiotropium over time. These data suggest that UMEC 125 μg and UMEC 62.5 μg would be appropriate doses to use in future clinical studies to further assess efficacy and safety of UMEC in COPD patients.

No assessments of COPD-related symptoms were included in this study. However, rescue salbutamol on an as-needed basis for breakthrough symptoms provided a secondary marker of symptom control. Treatment with UMEC reduced rescue salbutamol use compared with placebo at all doses and a dose ordering of reduction in salbutamol use was observed for UMEC QD doses, with UMEC 125 μg QD doses showing the greatest improvement. Rescue salbutamol use data are consistent with the observed improvements in lung function (i.e. the greatest reduction in use was observed with UMEC 125 μg QD, which had the greatest improvement in lung function parameters). Interestingly, the greatest reduction in salbutamol occurred with tiotropium. This may be due to the design of the study in that tiotropium was administered in an open-label fashion.

This was the first study to examine the PK of low-dose UMEC (15.6 μg and 31.25 μg QD and the BID dosing regimen) in patients with COPD. In spite of the large percentage of non-quantifiable data, increased UMEC systemic exposure (in terms of AUC and C_max_) was seen with lower doses. Median C_max_ occurred early for all UMEC doses. Systemic exposure was higher following QD dosing than BID dosing for the same total daily dose. Cross-study comparison of the steady-state data with previous studies of UMEC 62.5 μg to 1000 μg QD suggest that systemic exposure of UMEC was within the expected range [6,8].

The safety profile was comparable between treatment groups. Overall, AEs were higher with UMEC 125 μg QD dosing (18%) than placebo or the other active treatments; however, the individual AEs observed with the UMEC 125 μg QD consisted of reports of sinusitis, dysgeusia, nasopharyngitis, and headache. Headache was the most common AE across all treatments and did not appear related to active treatment. Although the AEs of nasopharyngitis, dysgeusia, and sinusitis could potentially be topical effects of LAMA treatment, these events occurred only with UMEC 15.6 μg and 125 μg QD dosing and so did not appear to be dose-related. Overall, UMEC was well tolerated across all dose regimens.

## Conclusion

In summary, UMEC QD statistically and clinically improved lung function over 24 hours in a study population that was representative of patients with moderate-to-severe COPD. UMEC was generally well tolerated, with dose- ordering over UMEC QD doses of 125–15.6 μg observed. Based on results from this study, the 62.5 and 125 μg doses were considered appropriate for subsequent clinical investigation.

## Endnotes

^a^ELLIPTA^TM^ is a trade mark of the GlaxoSmithKline group of companies.

^b^HandiHaler® is a registered trade mark of Boehringer Ingelheim International GmbH.

## Competing interests

All authors are employees of and hold stock in GlaxoSmithKline.

## Authors’ contributions

All listed authors meet the criteria for authorship set forth by the International Committee for Medical Journal Editors. AC, MB, JB and RM were involved in the conception and design of the study. AC, MB, and RM collected data. AC, MB, JB, RM and PS analysed and interpreted the data. All authors drafted and/or reviewed drafts of the manuscript and approved the final version for submission.

## Pre-publication history

The pre-publication history for this paper can be accessed here:

http://www.biomedcentral.com/1471-2466/14/2/prepub

## Supplementary Material

Additional file 1: Table S1 Final dose–response model parameters for trough FEV_1_ (mITT population). Click here for file

Additional file 2 Supplementary information. Click here for file

Additional file 3: Figure S1 Observed vs. population/individual predictions for UMEC QD and BID regimens (mITT population). **Figure S2.** Predicted trough FEV_1_ (L) vs. weighted residuals (Day 8) for UMEC QD and BID regimens (mITT population). Click here for file
