# Supplementary material for: Dose response of umeclidinium administered once or twice daily in patients with COPD: a randomised cross-over study
Source: BMC Pulm Med. 2014 Jan 6;14:2. doi: 10.1186/1471-2466-14-2 (PMC4029330; doi:10.1186/1471-2466-14-2)
Supplement: Additional file 1: Table S1 — Final dose–response model parameters for trough FEV1 (mITT population). [file 1471-2466-14-2-S1.docx]

**Additional File 1**

**Supplemental table**

Table S1 Final dose–response model parameters for trough FEV_1_ (mITT Population)

| **Parameters** | **Value for  Day 8 only** | **95% CI  (% RSE)** |
| --- | --- | --- |
|  |  |  |
| E_max_ (L) | 0.185 | 0.154–0.216 (9) |
| ED_50_ (C_50_ - mcg) | 37.4 | 17.8–57.0 (28) |
| S0 (L) placebo | 1.24 | 1.21–1.27 (1) |
| β-FEV_1_ MB-S0 | 0.691 (*p* <1e-010)^a^ | 0.65–0.73 (3) |
| ηE_max_ | 47% | 40 |
| ηED_50_ | 50% | 54 |
| ηS0 | 34% | 9 |
| Residual (ε) | Proportional 33% | 5 |

Note: 95% CI for fixed-effect parameter: ± 1.96 *SE.

^a^Wald Test.

β-FEV_1_ MB-S0, mean baseline trough forced expiratory volume in 1 second response; ε, residual variability; η, inter-subject variability; CI , confidence interval; ED_50_ (C_50_), dose that yields 50% of E_max_; E_max_, maximum predicted FEV_1_ response; RSE, relative standard error.
