# Supplementary material for: Dose response of umeclidinium administered once or twice daily in patients with COPD: a randomised cross-over study
Source: BMC Pulm Med. 2014 Jan 6;14:2. doi: 10.1186/1471-2466-14-2 (PMC4029330; doi:10.1186/1471-2466-14-2)
Supplement: Additional file 2 — Supplementary information. [file 1471-2466-14-2-S2.docx]

**Additional File 2**

**Supplementary information**

FEV_1ij_: FEV_1_ response (*j*) of individual i

E0: baseline FEV_1_ (L) (with placebo effect)

E_max_: maximum FEV_1_ response (L) in patient *i* reflecting the maximum difference in

response between placebo and treatment

Dose*_i_*: Dose of treatment (µg) in patient *i*

ED_50_: Potency – dose (µg) that produces 50% of E_max_

Θ(BID): fractional change for potency due to bid regimen

The E_max_ model was parameterised with a log normal distribution for parameters E0, E_max_ and ED_50_. This is consistent with physiological pharmacodynamic models as well as pharmacokinetic models to avoid improbable physiological values (e.g. ED_50_ less than 0). The parameters and confidence intervals were converted to a linear scale when reporting the output. In addition, if the parameter for fractional change for potency following QD versus BID dosing in the E_max_ model were insignificant (i.e. no difference in model fit diagnostics), subsequent model development focused on the QD data since the limited BID data would not add any more information. The η*_kj_* representing the inter-patient variability in model parameters were further assumed to be independent multivariate normally distributed, with mean 0 and variance (ω2) were estimated. Thus, _1_ *_j_* _η_ is a proportional individual baseline (placebo) shift and _2_ *_j_* _η_ is a proportional individual drug response shift.

For residual error in the population dose-response analysis, additive, proportional and combined additive and exponential error models were tested including influence of QD and BID regimens. ε is a random effect representing the residual error; that is, the difference between observed and predicted response for each individual using a combination of exponential and additive error. It was assumed to be normally distributed with mean zero and an estimated variance (σ2).

Inter-occasion variability in baseline FEV_1_ across different treatment periods was included in the model, taking advantage of the cross-over design in this study. The fit to the different potential dose-response models was compared using the objective function (OFV) calculated by NONMEM program. The OFV was proportional to the -2*log likelihood of the fits and was used to compare the goodness of fit of the various models in a sequential manner. A lower OFV value indicated a better fit. For example, the OFV value obtained after the step-linear model was compared to the reference step model with the difference between the two OFV being χ2 distributed. CIs on the parameter estimates were obtained from the usual approximate asymptotic covariance matrix of the estimates as well as from using parametric bootstrap modelling of final model.
