# Supplementary material for: Dose response of umeclidinium administered once or twice daily in patients with COPD: a randomised cross-over study
Source: BMC Pulm Med. 2014 Jan 6;14:2. doi: 10.1186/1471-2466-14-2 (PMC4029330; doi:10.1186/1471-2466-14-2)
Supplement: Additional file 3: Figure S1 — Observed vs. population/individual predictions for UMEC QD and BID regimens (mITT population). Figure S2. Predicted trough FEV1 (L) vs. weighted residuals (Day 8) for UMEC QD and BID regimens (mITT population). [file 1471-2466-14-2-S3.docx]

**Additional File 3**

**Supplementary figures**

**Figure S1 Observed vs. population/individual predictions for UMEC QD and BID regimens (mITT population).**

**
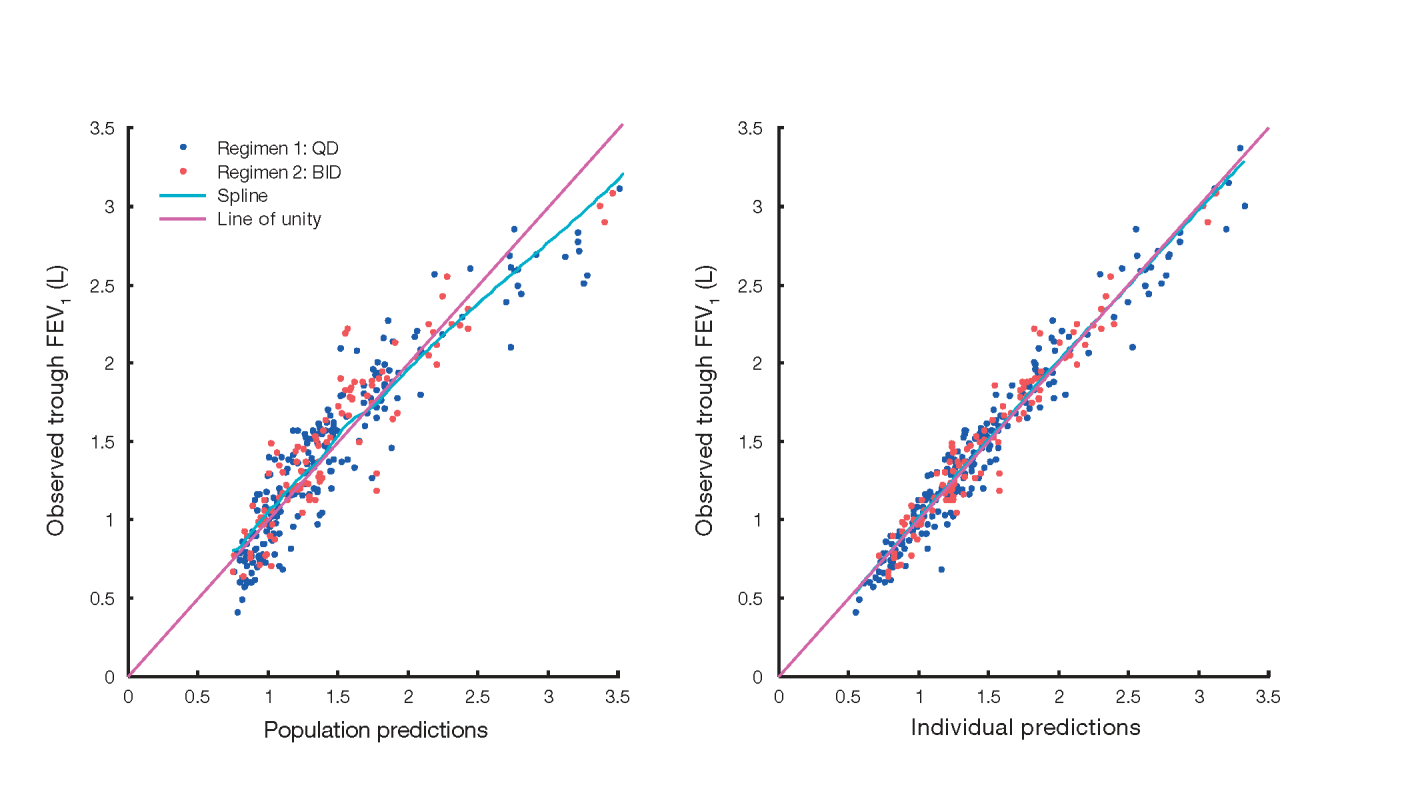
**

BID, twice daily; FEV_1_, forced expiratory volume in 1 second; QD, once daily.

**Figure S2 Predicted trough FEV_1_ (L) vs. weighted residuals (Day 8) for UMEC QD and BID regimens (mITT population).**

**
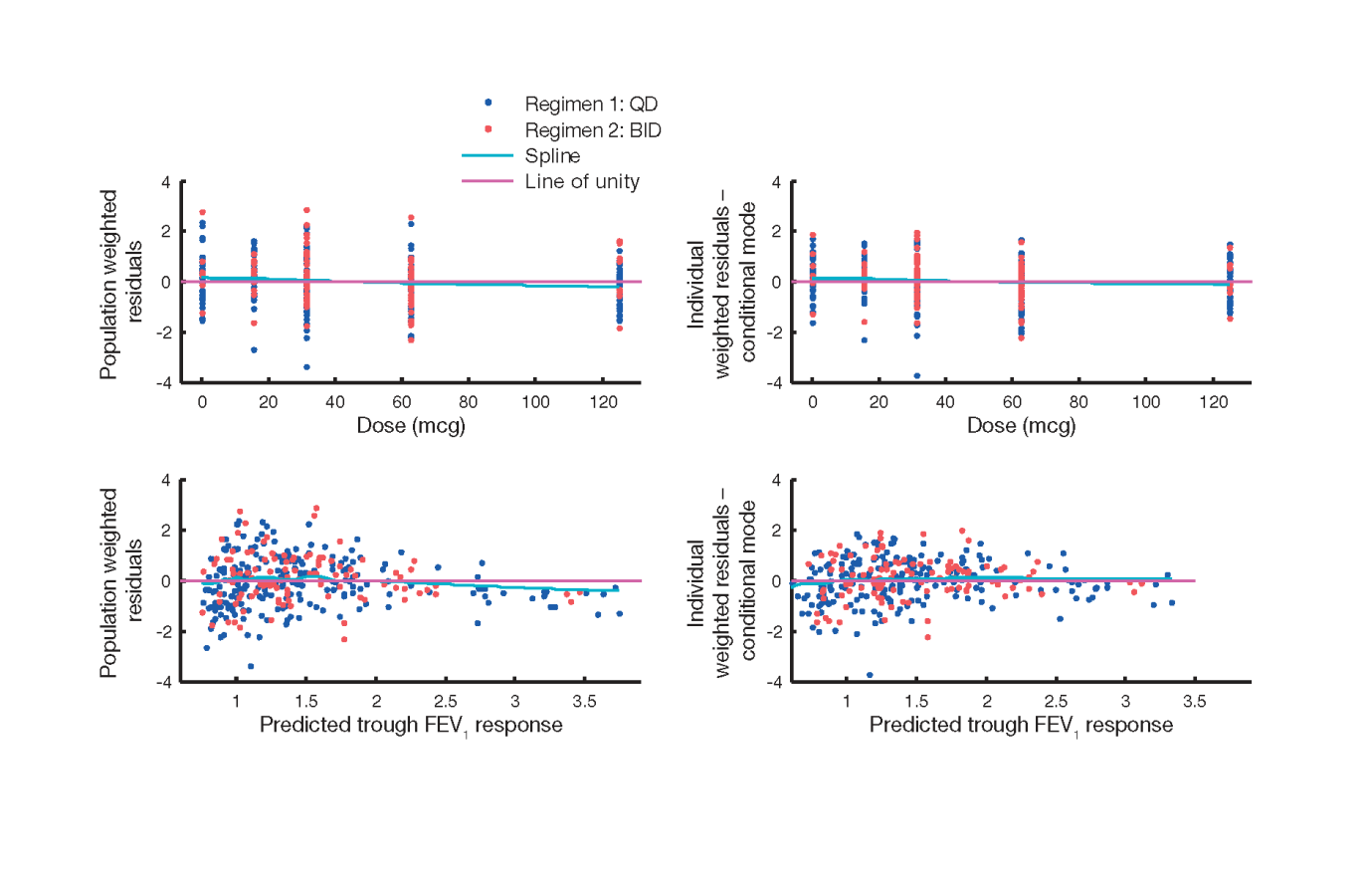
**

BID, twice daily; FEV_1_, forced expiratory volume in 1 second; QD, once daily.
